# The Role of Obstetrician/Gynecologists in the Management of Hepatitis C Virus Infection

**DOI:** 10.1155/2008/374517

**Published:** 2008-09-23

**Authors:** Bernard Gonik

**Affiliations:** Department of Obstetrics and Gynecology, School of Medicine, Wayne State University, Detroit, MI 48202, USA

## Abstract

Chronic infection with hepatitis C virus (HCV) is a major cause of liver disease-related death and is also the most frequent indication for liver transplantation in USA. Infected individuals can remain asymptomatic for 20 years or more, but they remain at risk for progressive liver disease. They also represent a potential source of infection for others. For reducing the future disease burden due to HCV, obstetrician/gynecologists and primary health care practitioners should be aware of the factors that promote HCV transmission: how to provide counseling and testing, and when specialist referral is needed.

## 1. INTRODUCTION

Hepatitis C virus (HCV) is transmitted mostly via infected blood or blood products [1] and is the most prevalent blood-borne virus in the United States, where an estimated 3.2 million people are chronically infected [2, 3]. Worldwide prevalence is estimated to be 170 million [4]. It is a single-stranded RNA virus of the family Flaviviridae. The viral genome shows great variability in nucleotide sequence owing to a high rate of mutation [3]. There are 6 major HCV genotypes and more than 50 subtypes [3]. This variation is believed to partly account for the ability of the virus to escape host immune defenses and sustain a chronic infectious state [3, 5].

The acute stage of HCV infection is only infrequently associated with symptoms of hepatitis such as anorexia, weakness, malaise, and jaundice [3]. It usually goes undiagnosed. Chronic infection is defined as infection persisting for longer than 6 months [3]. In most instances, HCV infection remains undetected until a chronic stage and is discovered during the course of testing for an unrelated condition or during blood donation [6]. It is estimated that acute infection develops into chronic infection in 60% to 85% of cases. Younger age, female sex, non-African-American race, and certain histocompatibility genes are all associated with improved spontaneous clearance of the virus and a lower likelihood of chronic infection [3].

HCV has been found in all regions of the world where it has been sought [4]. Approximately, 1.3% of the population of the United States is chronically infected [2]. Areas of higher prevalence include certain countries in the Far East, Mediterranean, Africa, and Eastern Europe. This worldwide reservoir of infection remains a potential source for new infections [4]. Most of the morbidity and mortality from HCV is the result of long-standing chronic infection. Of those chronically infected with HCV, approximately 5% of individuals aged <40 years and 34% to 58% of individuals aged ≥40 years develop cirrhosis of the liver 20 years after exposure and may ultimately die from end-stage liver disease or hepatocellular carcinoma [7]. In the United States, approximately 40% of chronic liver disease is HCV-related, which would suggest that 8 000 to 10 000 deaths each year are caused by HCV. In addition, HCV-associated end-stage liver disease is the most frequent indication for liver transplantation in adults [1].

The estimated incidence of new infections in the United States declined from 230 000 in the mid-1980s to 36 000 in 1996 [1]. The use of effective HCV tests has largely eliminated blood transfusion as a route of infection in the United States and other developed countries [1]. In addition, measures aimed at reducing the spread of human immunodeficiency virus (HIV) may be responsible for the observed reduction in new HCV infections in injection-drug users [8]. However, because liver disease is the result of long-standing infection, the burden of disease continues to rise. It is anticipated that there will be a 4-fold increase in the number of Americans at risk of chronic liver disease in 2015 compared with 1990 [8].

## 2. RISK FACTORS FOR HCV INFECTION

Persons at the greatest risk of HCV infection are those who have ever injected illicit drugs and those who have received blood transfusions or organ transplants before universal testing of donors was established. Also at risk are persons who received clotting factor concentrates. Lesser but still measurable risk factors for HCV include birth from an infected mother, sexual contact, accidental contamination during medical procedures, and household contact [1].

### 2.1. Parenteral transmission

Injection-drug users are at extremely high risk of acquiring HCV infection. It has been shown that the prevalence of HCV among older injectors (≥5 years of injection) is high (80% to 90%), whereas studies of young injectors (1–3 years of injection) in the last 10 years have shown that the cumulative infection rates have slowed [9]. Only 20% to 40% of young injectors are positive for HCV infection [10]. Injection-drug users become more rapidly infected with HCV than with other blood-borne viruses, possibly because of high levels of HCV chronicity in this population [11]. Injection-drug use accounts for most of the current transmission of HCV in the United States [1].

Persons who received blood transfusions or organ transplants before 1992 are considered to be at risk of having acquired HCV infection. In the United States, introduction of second-generation polyantigen testing from July 1992 largely eliminated the risk from donated blood and organs [1].

### 2.2. Perinatal transmission

The average rate of infection in infants born to infected mothers is 5% to 6%. The chance of infection has been shown to be greater with higher serum levels of HCV-RNA (17%) and in mothers coinfected with HCV and HIV (14%). HCV-RNA can be detected within 2 to 3 months of birth [1]. Children with HCV infection only rarely show symptoms or significant elevations in liver enzymes. Progression to severe liver disease is generally slower than in adults [12].

### 2.3. Sexual transmission

The relevance of sexual transmission to the epidemiology of HCV remains controversial. Epidemiologic surveys suggest that 20% of patients with recently acquired hepatitis C have no self-reported risk factor other than multiple sexual partners or an infected sexual partner [13]. However, studies of HCV transmission in stable monogamous couples show very low rates of infection—in some instances, below the detection limits of the study [14–18]. Most studies of couples have been cross-sectional in design and have investigated concordance rates for seropositivity. Only some of the studies included nucleotide sequencing; in these instances, HCV strains were sometimes found to be too dissimilar to be consistent with transmission between partners [19]. The rate of sexual transmission between monogamous heterosexual partners has been estimated to be between 0% and 0.6% annually [3, 19]. This low rate of HCV infection after many years of sexual exposure would suggest that the risk of sexual transmission of HCV is minimal. It is biologically plausible that rates of sexual transmission of HCV may be higher if exposure to blood occurs, and there is some supporting evidence for an association of acute HCV infection with genital ulcers or sexual activity at risk for lacerations of the anal epithelium [20, 21]. However, it is now widely believed that the observed association of HCV with high-risk sexual practices is mostly the confounding effect of unreported injection-drug use that is itself associated with high-risk sexual practices [1]. Many persons are unwilling to admit to a history of injection-drug use [22]. It should be emphasized that the association between markers of high-risk sexual practices and HCV infection is real, even if the underlying reasons are unclear. The Centers for Disease Control and Prevention (CDC) states that “although HCV is not efficiently transmitted sexually, persons at risk for infection through injection-drug use might seek care in STD [sexually transmitted disease] treatment facilities” [23].

### 2.4. Nosocomial transmission

In the United States and other industrialized countries, the use of standard (universal) precautions to prevent infection has eliminated most HCV transmission in health care settings. Three exceptions remain. First, the average incidence of seroconversion after needle stick or sharp exposure from an HCV-seropositive source has been estimated to be 1.8% (range 0% to 7%) [1]. One study showed a 1.2% incidence of seroconversion after injury from a hollow-bore needle but no instances of seroconversion from injuries with sharp objects or from contamination of mucosa or nonintact skin [24]. Second, despite stringent infection-control precautions, HCV outbreaks continue to occur in hemodialysis centers. Epidemiologic surveys show that the prevalence of seropositivity in patients in hemodialysis centers averages 10% and, in some centers, can be greater than 60% [1]. Third, during the last 15 years, there has been an increase of iatrogenic HCV transmission not related to dialysis [25]. It is being recognized with increasing frequency that HCV transmission is resulting from unsafe therapeutic injection practices, such as contaminated multiple-dose medication vials, saline bags from reinsertion of used syringes, and contaminated platforms for spring-loaded finger-stick devices used to monitor blood glucose in multiple patients. Interestingly, most of the instances occurred in developed countries, such as Western and Northern Europe, the United States, Australia, and Japan.

The CDC has hemodialysis and syringe/needle safety guidelines to prevent nosocomial HCV transmission in ambulatory healthcare settings [26]. The CDC recommends the following routine precautions for the care of all hemodialysis patients:

1. patients should have specific dialysis stations assigned to them, and chairs and beds should be cleaned after each use;
2. sharing among patients of ancillary supplies such as trays, blood pressure cuffs, clamps, scissors, and other nondisposable items should be avoided;
3. nondisposable items should be cleaned or disinfected appropriately between uses;
4. medications and supplies should not be shared among patients, and medication carts should not be used;
5. medications should be prepared and distributed from a centralized area;
6. clean and contaminated areas should be separated (e.g., handling and storage of medications and hand washing should not be done in the same area or an area adjacent to where used equipment or blood samples are handled).

The CDC recommends that all health care workers

1. use a sterile, single-use, disposable needle and syringe for each injection and to discard them intact in an appropriate sharps container after use;
2. use single-dose medication vials, prefilled syringes, and ampules when possible. Do not administer medications from single-dose vials to multiple patients or combine leftover contents for later use;
3. if multiple-dose vials are used, restrict them to a centralized medication area or for single patient use. Never reenter a vial with a needle or syringe used on one patient if that vial will be used to withdraw medication for another patient. Store vials in accordance with manufacturer's recommendations and discard if sterility is compromised;
4. do not use bags or bottles of intravenous solution as a common source of supply for multiple patients;
5. use aseptic technique to avoid contamination of sterile injection equipment and medications.

### 2.5. Household transmission

Nonsexual transmission of HCV between family members may infrequently occur, presumably via percutaneous or permucosal exposure to blood. HCV seropositivity concordance rates average 4% in nonsexual household contacts, including siblings and others [27, 28]. However, most such studies did not use confirmatory sequence analysis [28]. Moreover, many of the studies were conducted in areas where transmission from contaminated medical equipment is known to have occurred in the past, and this would alternatively explain HCV concordance in families [1].

## 3. ANTIVIRAL THERAPY

The standard therapy for chronic HCV infection is a course of weekly subcutaneous injections of pegylated interferon alfa (PEG-IFN) combined with once-daily oral ribavirin [12, 29]. Duration of treatment is 48, 24, or 12 weeks, depending on HCV genotype and other treatment prognostic factors. The principal goal of treatment is a sustained virologic response (SVR), defined as elimination of detectable virus during treatment and continued absence of virus 6 months after the end of treatment [12]. Long-term followup of patients in clinical trials has shown that an SVR usually presages long-term eradication of the infection and a marked reduction in the histologic and biochemical markers of liver disease [30, 31].

Pegylated interferon has a covalently attached water-soluble polymer of polyethylene glycol that improves the pharmacokinetic profile over that of standard interferon and allows for once-weekly dosing. Ribavirin is an antiviral drug that is effective against HCV only when combined with interferon. Although PEG-IFN has a greater efficacy than older anti-HCV therapies, it remains less effective against HCV genotype 1 than genotypes 2 and 3 [29]. Safety and efficacy of PEG-IFN have also been established in patients with HCV and compensated cirrhosis and in patients with HCV/HIV coinfection [32].

PEG-IFN can cause bone marrow suppression, and ribavirin can cause hemolytic anemia, resulting in the need for periodic hematologic testing during therapy. Thyroid function testing (thyroid stimulating hormone) is required because of the risk of autoimmune thyroiditis [29]. Emotional side effects of interferon are common [29], and patients need to be monitored for depression. Patients should be advised to report any sign or symptom of depression to their prescribing physician [32]. Ribavirin is contraindicated in pregnancy and in male partners of pregnant women. Thus pregnancy testing is required in women of childbearing potential immediately before and at monthly intervals during therapy. Women of childbearing potential and their male partners must use reliable forms of contraception if either partner is receiving PEG-INF/ribavirin, both during treatment and for at least 6 months after treatment has concluded [32].

For those individuals infected with the HCV virus, the CDC recommends treatment based on the level of clinical risk (Table 1). Treatment is generally recommended for patients who are at an increased risk of developing cirrhosis [29]. Such patients are characterized by detectable viremia, and a liver biopsy shows portal or bridging fibrosis and moderate inflammation and necrosis [3]. Some patients with milder disease than this also choose to be treated [3]. About one third of those with chronic HCV have a highly favorable prognosis and are unlikely ever to develop cirrhosis [33]. An informed choice between starting or deferring treatment is based on prognostic factors, the likelihood of treatment success, the potential for side effects, any relative or absolute contraindications, and on the patient's motivation [3, 34]. Patients deferring antiviral therapy should undergo a liver biopsy every 4 or 5 years to monitor disease progression [12].

## 4. SCREENING AND DIAGNOSIS

Obstetrician/gynecologists and other providers of primary health care services can screen for patients at risk of HCV infection, perform an initial diagnostic workup, and provide appropriate counseling to those infected or at risk of becoming infected. Routine screening of all adults is not recommended; instead, screening is focused on individuals with known risk factors for HCV infection [1]. These include the following:

1. persons who have injected illegal drugs, even if this took place in the remote past; this includes those who injected once or a few times [1]; many of these persons may not consider themselves to be drug users [22];
2. persons who received an organ transplant or blood transfusion before July 1992, when second-generation enzyme immunoassay (EIA) anti-HCV testing was introduced [1]; women who had cesarean sections before that date may have been at risk of infection via transfusion;
3. persons notified that they received blood from a donor who later tested positive for HCV [1];
4. persons who received clotting factor concentrates produced before 1987, when heat inactivation was introduced [1];
5. persons who have ever been on long-term hemodialysis [1];
6. persons with HIV infection [1];
7. patients with biochemical or clinical evidence of liver disease, for example, persistent elevations of alanine aminotransferase (ALT) [1, 29];
8. health care, emergency medical, and public service workers with needle stick injury or mucosal exposure to HCV-positive blood [1];
9. children born to HCV-infected mothers [1];
10. current sexual partners of HCV-infected persons. Although risk in this group is low, a negative test in the partner provides reassurance [12].

Patients can be screened for risk factors during any visit. An unexplained elevation of ALT may be caused by HCV, but many of those with chronic HCV infection have normal ALT. Signs and symptoms are sometimes present but are mostly nonspecific and include malaise, anorexia, and fatigue; these may be accompanied by low-grade fever and abdominal discomfort. Physical examination may reveal signs of liver disease such as hepatosplenomegaly, spider nevi, and palmar erythema [35]. Counseling and testing should be offered if a risk factor is identified [1]. Testing for HCV is not recommended for pregnant women without risk factors. Certain settings such as STD clinics and correctional facilities serve large numbers of patients at high risk for blood-borne infections, and it is especially important to screen for a history of injection-drug use in these settings [23].

## 5. TESTING PROCEDURES

EIA tests are inexpensive, reproducible serologic tests that detect anti-HCV antibodies. The version in current use (third-generation) has high sensitivity and specificity, making confirmatory testing unnecessary in many instances. EIA tests are recommended for initial testing of patients [3]. A positive test may be considered conclusive in patients with evidence of liver disease, such as abnormally high ALT, together with risk factors for infection [3]. Qualitative polymerase chain reaction (PCR) tests detect viral RNA with a greater sensitivity than the quantitative PCR tests that are used to measure viral load. Qualitative PCR tests can be used to confirm a positive EIA. A negative PCR result may require confirmatory immunoblot testing for anti-HCV. A repeat negative qualitative PCR result and confirmed anti-HCV test is suggestive of a spontaneously cleared acute infection. Patients with immune deficiencies or on hemodialysis may test false-negative for anti-HCV, and patients with autoimmune disease may test false-positive. A qualitative PCR test may be used as the primary test in these instances [3]. Infants born to infected mothers should be tested using EIA not earlier than 15 months of age to avoid the possibility of a false-positive result caused by maternal antibody. A PCR test can be performed during or after the first well-child visit at ages 1 to 2 months if an earlier result is required [3].

## 6. COUNSELING

Obstetrician/gynecologists and other primary care physicians can have an important role in counseling patients on how to slow disease progression and prevent transmission of the virus to others. Alcohol and HCV have independent and synergistic effects on the risk of cirrhosis [36]. Counseling patients to eliminate or at least reduce alcohol consumption may therefore be an effective primary care intervention for preventing progression to cirrhosis. Acceleration of progression to cirrhosis has been clearly established with heavy alcohol consumption, and the results of one study suggest that moderate consumption may be deleterious [12, 37]. The CDC recommends that patients with chronic HCV infection abstain from alcohol [1].

Counseling should also stress the importance of weight control. Both obesity and its accompaniment, fatty liver disease, are associated with progression of HCV-associated liver disease. Thus weight reduction for those with body mass index >25 kg/m^2^ may be especially beneficial in persons with chronic HCV infection. Hepatitis A and B vaccinations are recommended for all persons with chronic HCV infection because of the increased risk of complications from coinfection, including fulminant hepatitis [12, 38].

Persons with HCV need reliable advice on how to avoid transmitting the infection to others, and uninfected persons need to know how to reduce the risk of infection. Health care professionals should routinely obtain a history that inquires about use of illegal drugs and evidence of high-risk sexual practices (e.g., multiple sex partners or a history of STDs) [1]. Knowing the risk factors and cross-checking the patient's history for these can have a positive impact on prevention of HCV transmission. Guidance in obtaining a sexual history is available from the curriculum provided by CDC STD/HIV Prevention Training Centers (http://www.stdhivpreventiontraining.org/) [23].

Current injection-drug users, regardless of HCV status, should be advised to stop using and injecting drugs and to enter and complete substance abuse treatment. If they continue to inject, they must use only sterile needles and syringes from a reliable source and never reuse or share drug preparation equipment, plus they must safely dispose of used materials [1].

Primary prevention of illegal drug injection will eliminate the greatest risk factor for HCV infection [1]. A study showed that injection-drug users, even before they become identified as high-risk by prevention services, may engage in more high-risk practices than individuals who remain nonusers [39]. Thus identifying risk factors for starting injecting could increase the opportunities to intervene and, in turn, cut transmission of HCV. Counseling and education to prevent the initiation of drug-injection or high-risk sexual practices is important, especially for adolescents [6].

Obstetrician/gynecologists should be aware that although consistent data are lacking on the extent to which sexual activity contributes to HCV transmission, persons with multiple sex partners are at risk for STDs, including HIV, human papillomavirus, hepatitis B virus, syphilis, gonorrhea, and *Chlamydia* [1].

Obstetrician/gynecologists should counsel persons at risk for STDs with regard to what they can do to minimize their risk of becoming infected or of transmitting infectious agents to others. Data on long-term sexual partners with no other risk factors suggest that the rate of sexual transmission of HCV is very low. Guidelines state that HCV-positive individuals do not need to change their sexual practices and that barrier forms of protection, such as condoms, are not required [12, 23]. Although monogamous sexual relationships carry a low risk of HCV transmission, the risk is higher in HCV-infected individuals with multiple sexual partners or in short-term relationships. In persons at risk for sexually transmitted diseases, guidelines advise the correct use of latex condoms during all sexual encounters [1].

HCV-positive women do not need to avoid pregnancy or breastfeeding [1]. However, prospective and expectant mothers concerned about HCV should be advised that

1. routine testing for HCV is not recommended for pregnant women unless there is a known risk factor [1];
2. approximately 5% of infants born to infected mothers become infected at birth, and there is no known treatment that can prevent this from happening. The choice of cesarean versus vaginal delivery does not appear to modify the risk of transmission [1];
3. infants and children with HCV are less likely than adults to have symptoms, and liver disease develops more slowly. Spontaneous clearance of HCV is more common in children than in adults [1, 12];
4. available evidence suggests that breastfeeding does not transmit HCV, although mothers should consider avoiding breastfeeding if the nipples are cracked or bleeding [1].

## 7. CONCLUSION

Hepatitis C and its associated liver disease is a global health problem that is likely to increase in coming decades because of a greater number of persons with long-standing chronic infection. Antiviral therapies have improved in recent years and can eradicate HCV infection in many individuals. Because of the subclinical nature of chronic HCV, identification of infected individuals who might benefit from therapy is central to the overall management of the disease. Obstetrician/gynecologists and other health care providers should be able to screen patients for risk factors and offer counseling and testing. All patients testing positive for HCV are potential candidates for antiviral therapy and should therefore be referred to specialists. Alcohol avoidance and weight control may be effective in delaying or preventing the onset of cirrhosis in those who choose to defer antiviral therapy.

Screening and counseling guidelines for hepatitis C that emphasize the role of nonspecialists include CDC guidelines on sexually transmitted diseases at http://www.cdc.gov/std/treatment/default.htm, which has a section on hepatitis C, and guidelines from the World Health Organization at http://www.who.int/csr/disease/hepatitis/whocdscsrlyo2003/ en/. Patient education leaflets on hepatitis C can be located at the US Department of Veterans Affairs at http://www.hepatitis.va.gov/vahep?page=prtop02-00-rr#S16X and at the National Library of Medicine Medline Plus at http://www.nlm.nih.gov/medlineplus/hepatitisc.html.

Screening for HCV risk factors take place at any health care visit. Obstetrician/gynecologists should note that patients in certain settings, such as STD treatment clinics, may be at increased risk of HCV infection. Sexual transmission of HCV appears to occur only rarely in stable monogamous couples, and precautions to prevent sexual transmission are not needed in these circumstances. HCV testing need not be offered to pregnant women with no identifiable risk factor; however, prenatal visits are a good opportunity to screen for HCV risk factors and to offer testing and counseling where appropriate. This is in part because health care visits may be more frequent than at other times in a woman's life and also because of the need to follow up children born to infected mothers.

## Figures and Tables

**Table 1:** Treatment groups for patients with hepatitis C virus (HCV) [1].

**Individuals recommended for treatment**
(i) Patients with persistently elevated alanine aminotransferase (ALT) levels
(ii) Patients with detectable HCV ribonucleic acid
(iii) Patients with a liver biopsy indicating either portal or bridging fibrosis or at least moderate degrees of inflammation and necrosis
**Individuals for whom treatment is unclear**
(i) Patients with compensated cirrhosis (without jaundice, ascites, variceal hemorrhage, or encephalopathy)
(ii) Patients with persistent ALT elevations but with less severe histologic changes (i.e., no fibrosis and minimal necroinflammatory changes) (In these patients, progression to cirrhosis is likely to be slow, if at all; therefore, observation and serial measurements of ALT and liver biopsy every 3–5 years is an acceptable alternative to treatment with interferon)
(iii) Patients <18 years of age or >60 years of age (note that interferon is not approved for patients younger than 18 years)
**Individuals for whom treatment is not recommended**
(i) Patients with persistently normal ALT values
(ii) Patients with advanced cirrhosis who might be at risk for decompensation with therapy
(iii) Patients who are drinking excessive amounts of alcohol or who are injecting illegal drugs (treatment should be delayed until these behaviors have been discontinued for ≥6 months)
(iv) Persons with major depressive illness, cytopenias, hyperthyroidism, renal transplantation, evidence of autoimmune disease, or who are pregnant
